# Hepatoid adenocarcinoma of the lung with PD-L1 expression and the response to anti-PD-1 therapy: a case report

**DOI:** 10.3389/fonc.2023.1257931

**Published:** 2023-11-23

**Authors:** Yan Li, Lili Liu, Dongfeng Wang, Xiaojing Tan, Yanmin Sui, Honglan Yang

**Affiliations:** ^1^ Department of Oncology, Dongying People’s Hospital, Dongying, Shandong, China; ^2^ Department of Pathology, Dongying People’s Hospital, Dongying, Shandong, China; ^3^ Department of Chest Surgery, Dongying People’s Hospital, Dongying, Shandong, China

**Keywords:** hepatoid adenocarcinoma of the lung, alpha-fetoprotein, anti-PD-1 therapy, brain metastases, case report

## Abstract

Hepatoid adenocarcinoma of the lung (HAL) is extremely rare; a standardized treatment strategy for HAL has not been established. The prognosis of patients with unresectable HAL is extremely poor. Here, we reported a 64-year-old male patient with unresectable alpha-fetoprotein-producing HAL who showed moderate harboring programmed death ligand 1 (PD-L1) expression and no targetable driver mutations. The patient was treated with brain radiotherapy, multiple lines of chemotherapies, and PD-1 inhibitor and achieved a survival rate of 9 months. The patient finally died because of the progression of brain metastasis. The case report provides valuable information for the treatment strategy development of advanced HAL patients and reminds us of the therapeutic particularity of brain metastasis.

## Case presentation

**Figure 1:**
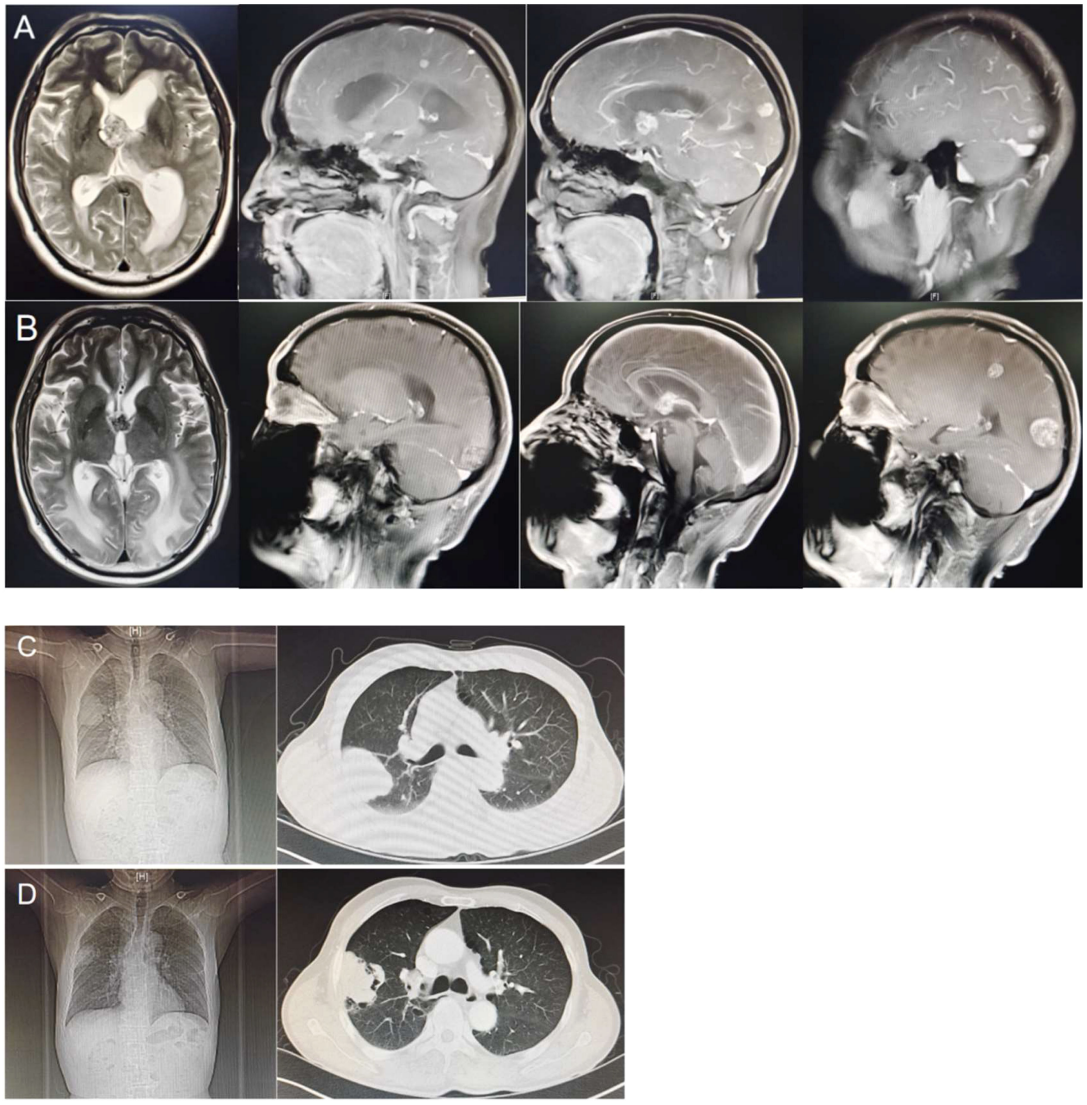
**(A)** Magnetic resonance imaging showed ventricular occupancy with hydrocephalus (15 January 2022). **(B)** Magnetic resonance showed leukoedema even worse, brain metastasis overall progress, and meningeal signal enhancement than before (15 September 2022). **(C)** A computed tomography scan showed a soft tissue mass in the upper lobe of the right lung (18 January 2022). **(D)** Computed tomography scan showed the primary lesion of the right lung was smaller than before and accompanied by cavity formation (05 September 2022).

**Figure 2:**
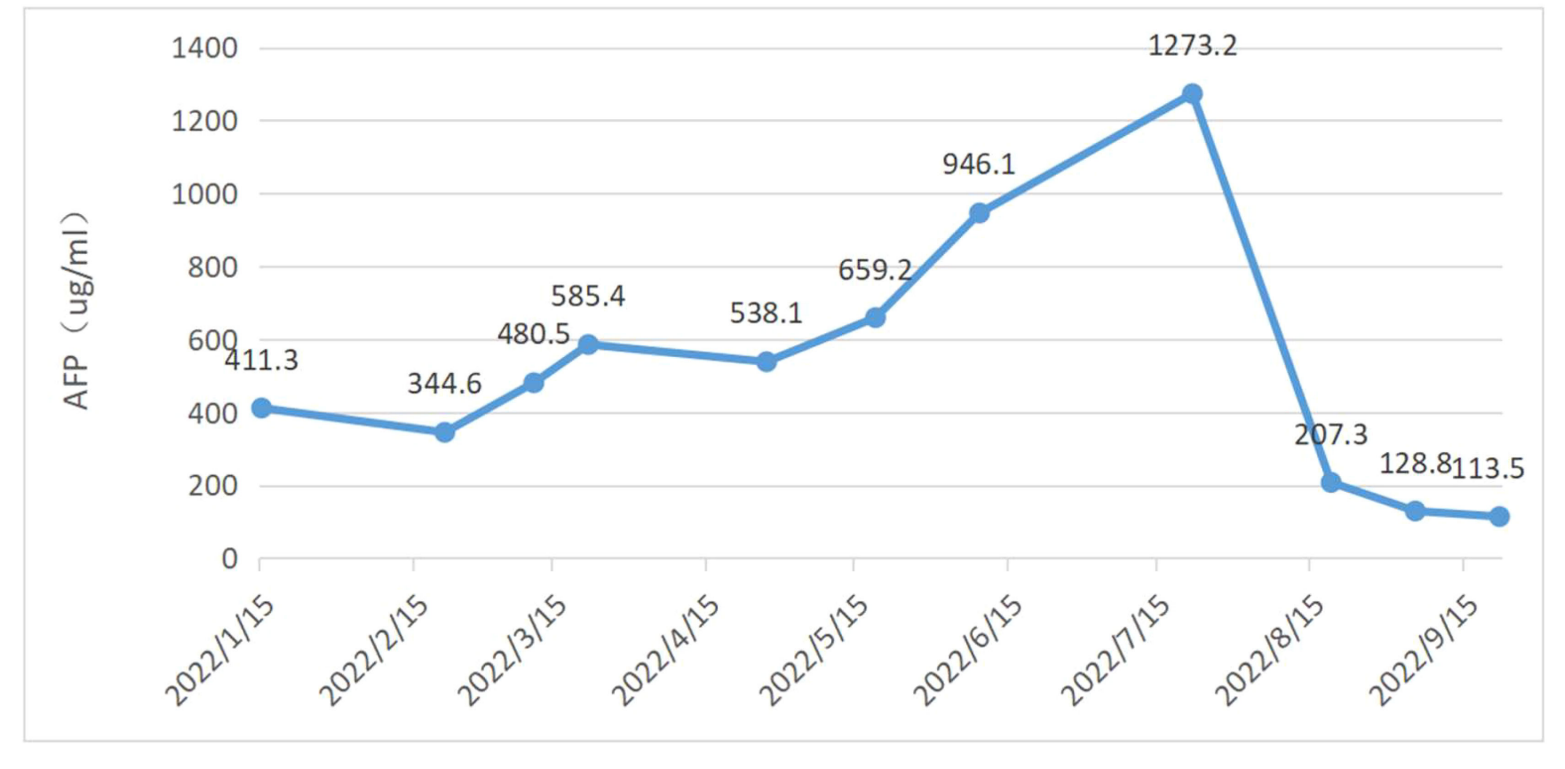
Kinetics of serum AFP level and timeline of therapies.

**Figure 3:**
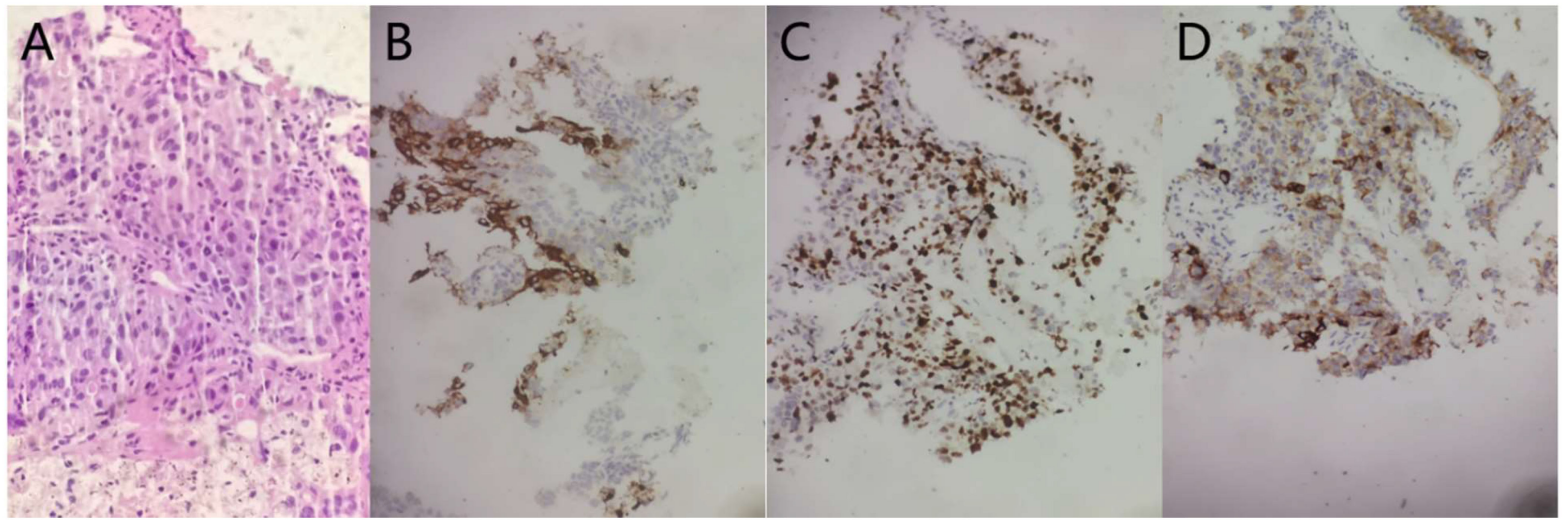
The pathology of the puncture biopsy of the right upper lung showed poorly differentiated carcinoma, and HAC was considered first by morphology (**A** HE staining morphology, original magnification, ×40). The lung tumor cells were weakly positive for Syn (**B**, immunohistochemical (IHC), original magnification, 20). The lung tumor cells were focal positive for CgA (**C**, IHC, original magnification, ×20). The lung tumor cells were 50% positive for Ki67 (**D** IHC, original magnification, ×20).

A 64-year-old man with a 40-year smoking history (Smoking Index 800) visited the Department of Neurology on 14 January 2022, because of a headache without obvious signs except for limb balance dysfunction. The magnetic resonance imaging (MRI) showed ventricular occupancy with hydrocephalus (**Figure 1A**). Tumor markers showed AFP 411.3 ng/mL (**Figure 2**), CEA 14.8 ng/mL, CYFRA21-1 6.1 ng/mL, NSE 22.2 ng/mL. A chest computed tomography (CT) scan demonstrated a soft-tissue mass in the upper lobe of his right lung, with metastasis of the right lung, the mediastinal lymph node, and the right hilar lymph node. The right upper pulmonary artery was invaded (**Figure 1C**). The pathology of the puncture biopsy of the right upper lung showed poorly differentiated carcinoma, and HAC was considered first by morphology. Immunohistochemistry showed Syn (weak +), CgA (focal +), CK7 (+), Ki67 (+, 50%), CK20 (focal +); P63 (−), NapsinA (−), CK5/6 (−), NSE (−), TTF-1 (−), and LCA (−) (**Figure 3**). Genomic testing showed moderate harboring PD-L1 expression (12.21Ct) and no targetable driver mutations (EGFR, ALK, ROS 1, BRAF, KRAS, ERBB 2, MET, RET, NTRK 1, NTRK2, and NTRK 3).

Radiotherapy was first given to reduce the neurological symptoms. On 11 March 2022, a chest CT examination for left chest pain, cough, and asthma revealed a double pulmonary artery embolism and pulmonary infarction in the left upper lung lobe. A color ultrasound found thrombosis in the right calf intermuscular vein. After that, anticoagulant treatment and pain relief treatment were given. According to the NCCN Guidelines (2022), systemic chemotherapy is the first-line therapeutic regimen, which can be combined with immunotherapy. Six cycles of sintilimab (a kind of PD-1 inhibitor) + pemetrexed + carboplatin treatment were started on 17 March 2022. During this period, the disease continued to progress slowly, and secondary reduction of leukocytes and platelets occurred repeatedly, but the symptoms continued to improve. The disease progressed significantly in July 2022. Because of a pulmonary infarction, the patient was not suitable for antivascular-targeted therapy and therefore had fewer opportunities for the following treatment. Immunotherapy is recommended in second-line therapy, and only chemotherapy is not expected to work well. Considering the slow progression during first-line treatment, the second-line chose docetaxel chemotherapy based on immunotherapy for two cycles. Granulocyte deficiency occurred during this period. The serum AFP level dramatically declined, and the imaging examination showed that pulmonary lesions and metastatic lymph nodes were decreased, but brain metastases were slightly larger with leukoedema and meningeal signal enhancement (**Figures 1B, D**). Neurological symptoms such as headache, memory decline, and equilibrium disorder continue to worsen. We first suspected that brain metastasis worsened, but meningeal metastasis was also possible, and tumor treatment-induced brain damage was also not excluded. This is also the problem that many patients with brain metastases ultimately need to face. However, the patient refused lumbar puncture for cerebrospinal fluid testing, as well as intrathecal injection. Temozolomide chemotherapy was attempted, but it was not tolerated due to nausea. His condition continued to worsen until he was discharged home after becoming in a coma and eventually died in October 2022.

## Discussion

Patients with early HAL can achieve relatively long-term survival after radical resection; therefore, early diagnosis and timely surgery contribute to improving overall survival. Patients with unresectable HAL have a poor prognosis. The brain is one of the most common metastatic sites of lung cancer, which is mostly insensitive to systemic antitumor therapy and has limited local therapeutic effects, and most patients die from brain/meningeal metastasis (1–4). Muroyama reported a HAL patient with solitary brain metastasis survived more than 19 months after resection of the brain tumor and chemotherapy (5). Chen reported a patient with driver gene mutations also achieved longer survival after EGFR-TKI target therapy (6). The patient we reported was at stage IV and lived for 9 months. Reviewing the entire treatment process, we got some experience and lessons. After the detection of brain metastasis, the primary lesion could be traced and diagnosed as soon as possible, timely radiotherapy reduced the symptoms, and anti-PD-1 treatment provided in the first line improved the efficacy. But there were also regrets, no early intervention was conducted for the hypercoagulable state of advanced tumors, so the emergence of pulmonary embolism and pulmonary infarction caused limited antivascular targeted therapy. For lung cancer patients with negative mutations of the driver gene, antivascular targeted therapies such as bevacizumab and anlotinib have an obvious dominant position, especially in patients with brain metastases. Furthermore, without cerebrospinal fluid testing and intrathecal injection, we could not exactly discriminate and cure brain metastasis, meningeal metastasis, and sometimes tumor treatment-induced brain damage. In addition, we noticed that the serum AFP level was not consistent with imaging and clinical manifestation. Considering the possibility of combining other cell types, one more biopsy was recommended, but the lineal kin refused. This is also a regretful part throughout the course of the patient.

## Conclusions

In summary, HAL lacks a standard treatment regimen and usually consults adenocarcinoma. The prognosis for unresectable patients is extremely poor. This year, with the update of the cancer treatment concept, Targeted therapy, anti-PD-1 therapy, and antiangiogenic therapy have been added to traditional treatment options (such as surgery, radiotherapy, and chemotherapy). Early combination therapy may improve the disease control rate (DCR). Timely local treatment can help reduce symptoms. The management of VTE is very important and sometimes affects the treatment opportunity and prognosis of the whole condition. Finally, the therapeutic goal of HAL is to reduce symptoms and tumor load and prolong survival.

## Data availability statement

The original contributions presented in the study are included in the article/supplementary material. Further inquiries can be directed to the corresponding author.

## Ethics statement

Written informed consent was obtained from the individual(s) for the publication of any potentially identifiable images or data included in this article.

## Author contributions

YL: Writing – original draft, Writing – review & editing, Data curation, Methodology, Project administration. LL: Data curation, Writing – review & editing. DW: Resources, Writing – review & editing. XT: Formal Analysis, Methodology, Writing – review & editing. YS: Methodology, Writing – review & editing. HY: Methodology, Writing – review & editing.
